# What Is Apoptosis and Why Is It Inhibited by the Most Important Tumor Suppressor (p53)?

**DOI:** 10.3390/ijms262110505

**Published:** 2025-10-29

**Authors:** Razmik Mirzayans

**Affiliations:** Department of Oncology, Cross Cancer Institute, University of Alberta, Edmonton, AB T6G 1Z2, Canada; razmik.mirzayans@cancercarealberta.ca

**Keywords:** p53, p21, WIP1, apoptosis, anastasis, senescence, therapy resistance, polyploid giant cancer cells, PGCCs

## Abstract

Anticancer strategies targeting the DNA damage response are largely centered on a number of false hypotheses. For example, engaging apoptosis in solid tumors is universally assumed to represent a tumor suppression response. But what is “apoptosis”, really? Time-lapse microscopy and other single-cell assays have revealed that engaging apoptosis in solid tumor cells is accompanied by anastasis, the homeostatic process of cell recovery from late stages of apoptosis, even after the formation of apoptotic bodies. Furthermore, apoptotic cells secrete a variety of prosurvival factors that contribute to overall tumor repopulation. Not surprisingly, numerous clinical studies reported since the 1990s have demonstrated that increased apoptosis in solid tumors is associated with cancer aggressiveness rather than representing a favorable clinical outcome. Another major false hypothesis pertains to the role of wild-type p53 in regulating apoptosis. Several recent articles addressing the challenges that have been encountered in implementing p53-based cancer therapies assume that p53 is pro-apoptotic. This assumption, which has become an almost indisputable fact, is shocking given that by mid-2000s it was already well established that p53 serves to inhibit apoptosis through upregulating ~40 anti-apoptotic proteins. The complexity of cancer cell response to therapeutic agents is discussed herein with a focus on the significance of p53-p21^WAF1^ signaling in suppressing the apoptosis–anastasis tumor repopulation pathway.

## 1. Introduction

Sarabjot Pabla has recently published an online (LinkedIn) article entitled “Contrarian Thinking in Bioinformatics: Unlocking Breakthroughs by Challenging Assumptions” in which he states that some of the major advances in science have not come from following the obvious path but from asking whether the current way of doing things is exactly what’s holding us back [1]. “Contrarian logic doesn’t mean rejecting consensus for its own sake. It means re-examining defaults, finding blind spots, and testing counterintuitive ideas that lead to better answers,” stated Pabla, a clinical and research bioinformatics expert [1].

**Figure 1:**
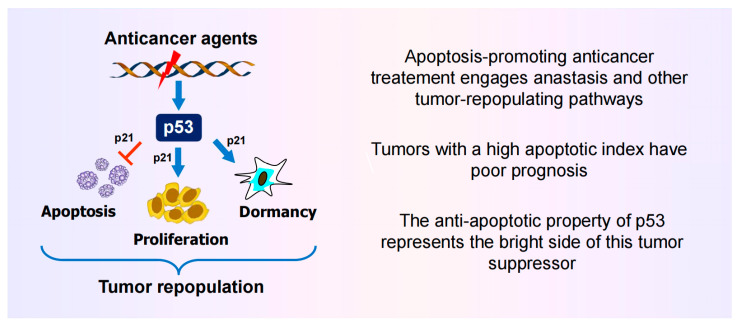
A highly simplified graphic summary and the main take-home messages of the studies reviewed herein. Arrows indicate stimulation and the T-shaped line indicates inhibition.

The current review presents “contrarian thinking” based on solid preclinical and clinical data regarding regulated (or programmed) cell death and p53 function (Figure 1). I prefer to use “false hypothesis” rather than “contrarian thinking” for highly simplistic and outdated (1990s) assumptions that have derailed cancer research for decades and, unfortunately, continue to do so. Some of these false hypotheses have been discussed ([2,3,4]; also see Appendix A).

Over the past three decades, apoptosis and other modes of regulated cell death, together with the transcription regulators p53 and p21^WAF1^ (p21), have been among the most extensively studied and highly reviewed fields in the context of cancer progression and therapy. Despite this, the following three fundamental questions still remain: (i) What is apoptosis? (ii) How is apoptosis influenced by p53 signaling? and (iii) What are the reasons for repeated failures in implementing novel anticancer strategies? The intention of the current article is to shed some light on these questions.

Please note that the discoveries highlighted herein are made with cell types (e.g., solid tumor cells) that predominantly undergo dormancy (active sleep) under stressful conditions. The situation might be quite different for other cell types such as lymphocytes and thymocytes that are programmed to be eliminated via apoptosis during negative selection or in response to stress.

## 2. Is Apoptosis a Tumor Suppression Mechanism?

### 2.1. Precision Oncology Targeting Apoptosis: Reality or False Promises?

“Precision oncology is inspirational. What doctor or patient would not want to harness genetics to tailor a therapy to an individual? But traveling back in a time machine is also inspirational. Who would not want to wind back the clock to remove their cancer before it spreads? In both cases, however, as of 2016, the proposal is neither feasible, cost-effective nor assured of future success. Yet in only one of these cases does the rhetoric so far outpace the reality that we risk fooling even ourselves” [5].

These remarks were made by Vinay Prasad in a Perspective article entitled “The precision-oncology illusion” that was published in *Nature* a decade ago [5]. While numerous authors have argued that precision oncology is not an illusion, a handful of other authors have highlighted compelling preclinical and clinical data that strongly support Prasad’s conclusion and have referred to personalized/precision oncology as “failed medicine” or (empty) promises that remain to be fulfilled [6,7,8,9,10,11,12,13,14,15,16,17,18].

There is no doubt that a small fraction of cancer patients do respond exceptionally well to radiotherapy, chemotherapy, and other mainstream treatments [19]. For the majority of cancer patients, however, particularly for patients with metastatic disease, traditional or targeted (precision) anticancer treatment, which is designed to eradicate solid tumors, has proven to cause more harm than benefit. In fact, as pointed out by Frank Arguello, the life expectancy of patients with esophageal cancers, for example, has not improved significantly over the span of a century (reviewed in [2]). This is perhaps not surprising, given that a presumed friend (apoptosis) has turned out to be the worst enemy in cancer therapy, fueling the oncogenic process, rather than promoting cancer cell demise (“suicide”) [20,21,22,23,24,25,26,27,28,29,30,31,32] (also see below).

The dark side of apoptosis in cancer therapy has been extensively discussed by us [2,3,4] and others [20,21,22,23,24]. Some key discoveries are outlined below to illustrate the need for new directions in the management of solid tumors, focusing on apoptosis-suppressing strategies.

### 2.2. The Apoptosis–Anastasis Tumor-Repopulating Pathway

**Figure 2:**
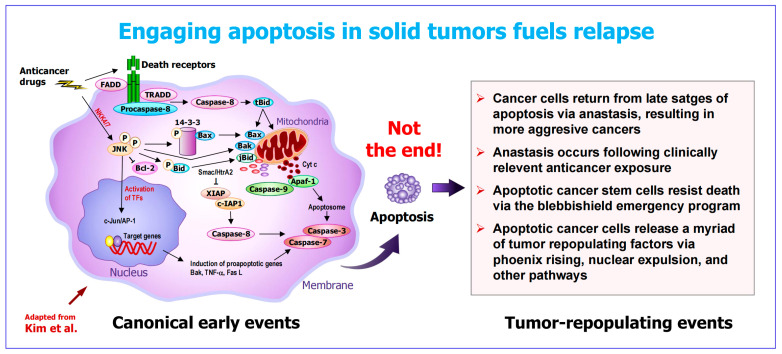
Early (canonical) and late (pro-survival) events after triggering apoptosis in solid tumors. For details, see text and [4]. The illustration of the canonical component is adapted from Kim et al. [34] and is meant to present a general molecular event after engaging apoptosis. It is by no means a comprehensive illustration, and some molecules are offset for clarity. For detailed description of intrinsic and extrinsic pathways of apoptosis, please consult the recent review by the Nomenclature Committee on Cell Death [35].

The process known as apoptosis has two components (Figure 2): The canonical component (traditionally referred to as “apoptosis”) accompanied by anastasis. The former involves the activation of initiator caspases, mitochondrial outer membrane permeabilization (MOMP), release of cytochrome c and other apoptogenic factors from the mitochondria into the cytoplasm, activation of apoptotic proteases (executioner caspases), nuclear fragmentation and formation of apoptotic bodies [33,34,35]. This is followed by anastasis, the natural phenomenon by which cells return from late stages of apoptosis and other forms of regulated cell death [23,24,28,29,30,31,32]. Thus, the formation of apoptotic bodies, which is traditionally labeled as “apoptosis” (presumably implying cell demise), is not the end of the apoptosis–anastasis journey.

Cancer cells undergoing anastasis exhibit increased invasiveness, metastatic potential, and therapy resistance when compared to non-anastatic (bulk) cancer cells [32]. The cell adhesion protein cadherin 12 (CDH12) [36], cIAP2/NFκB [37], and p38 MAPK signaling [38] are implicated in anastasis-driven tumor angiogenesis and metastasis.

The cell surface expression of CD24 has been recently reported to be preferentially enriched in anastatic cancer (melanoma) cells that exhibit tumorigenic properties [39,40]. According to Vasileva et al. [39], even CD24-positive cancer cells that display various cell “death” indicators are able to recover and form large colonies under 3D culture conditions. These indicators included trypan blue staining (a marker of transient loss of cell membrane integrity), annexin V staining (a marker of phosphatidylserine externalization as well as loss of cell membrane integrity), nuclear fragmentation, and cell detachment from the culture surface.

### 2.3. Other Apoptosis-Related Tumor-Repopulating Pathways

In addition to anastasis, various other pro-survival pathways are associated with cancer cells undergoing apoptosis. These include phoenix rising [41,42], nuclear expulsion [43], senescence reversal [44], and the blebbishield emergency program (observed in cancer stem cells) [45,46] (for details, please see our recent reviews [2,3,4]).

### 2.4. Increased Apoptosis in Solid Tumors Is Linked to an Unfavorable Clinical Outcome

Clinical studies reported since 1996 [47] have established that increased apoptosis in solid tumors is associated with cancer aggressiveness and poor patient outcomes (e.g., [48,49,50,51,52,53,54,55,56,57,58,59,60,61,62,63,64]). Some of these studies involved a large cohort of cancer patients. For example, the meta-analysis reported by Yang et al. in 2018 [54] was performed with 3091 breast cancer cases.

### 2.5. Take-Home Messages

Collectively, these pre-clinical and clinical observations challenge the popular hypothesis that apoptosis might be a tumor suppression mechanism. They also underscore the danger of relying on molecular, biochemical, and morphological manifestations of apoptosis as a marker of cancer cell death and call for revisiting thousands of articles that have used the terms “apoptosis” and “death” interchangeably.

## 3. Apoptotic Cancer Cells Promote Tumor Diversity and Heterogeneity

**Figure 3:**
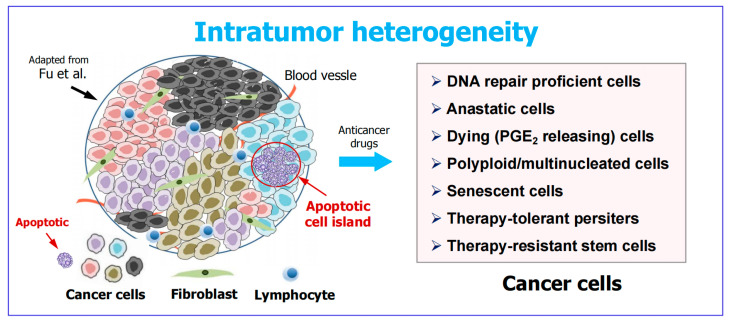
Left: Complex heterogeneity within a solid tumor (adapted from Fu et al. [67]). A region enriched with apoptotic cells is marked. Such “apoptotic cell islands” stimulate the proliferation of surrounding cancerous cells, which is in part mediated by oncogenic caspase 3 [41,42]. Right: Examples of cancer cell types that can promote tumor repopulation post therapy. A subset of cancer cells increase their p21 protein levels in response to treatment which is “just right” to enable them to temporarily halt their cell cycle, repair their genome and resume proliferation [68]. Anastasis refers to the natural process of cell recovery from late stages of regulated cell death [23,24]. Dying cells (e.g., through apoptosis) release a panel of pro-survival factors, including prostaglandin E_2_ (PGE_2_) via the phoenix rising pathway [20,25]. Polyploid/multinucleated giant cancer cells (PGCCs), senescent cancer cells, and therapy-tolerant cancer cells are three cell subgroups that enter a state of transient dormancy (active sleep) post-therapy [3,69]. Cancer stem cells undergoing apoptosis resist destruction (phagocytosis) by fusing their apoptotic belbs to form a blebbishield [26,45].

In response to moderate levels of stress, such as clinically relevant chemotherapy exposure, virtually all apoptotic cancer cells are known to undergo anastasis when determined in tissue culture studies (see, e.g., [4] and the video in [65]). The situation might be quite different in the tumor microenvironment, where cancer cell fate is influenced not only by the interplay between different cell types but also by a myriad of molecules released from dying cells ([66,67,68,69] and Figure 3). Thus, to what extent apoptotic cancer cells that display the “eat me” signals (e.g., phosphatidylserine exposure) will be eliminated by the immune system remains unknown.

Irrespective of what proportion of apoptotic cancer cells will be destroyed, such cells are known to “sacrifice themselves at the altar of heterogeneity” via “treacherous apoptosis” [22]. This phenomenon refers to the presence of densely populated caspase 3-positive cells within an individual tumor (apoptotic cell islands) that fuel the proliferation and survival of cancerous and non-cancerous cells nearby, thus creating a diverse tumor population [66].

At first glance, it would appear that targeting (inhibiting) treacherous apoptosis together with signaling pathways associated with anastasis might improve the outcome of cancer therapy. This possibility is unlikely to be tenable given that a number of apoptosis-unrelated responses are known to contribute to intratumor heterogeneity [67,68,69,70,71]. These include cancer cell dormancy (a potential characteristic or mechanism underlying minimal residual disease) [70], as well as extrinsic factors such as angiogenesis, hypoxia, oxidative stress and acidosis [71].

## 4. Intratumor Heterogeneity: A Well-Established (Yet Widely Overlooked) Obstacle in Cancer Therapy

The impact of tumor heterogeneity on implementing the various branches of precision oncology (e.g., strategies targeting p53, p21, DNA-damage response, regulated cell death, etc.) is becoming increasingly appreciated (e.g., [72,73,74,75,76,77,78,79,80,81,82]). But this knowledge is not new!

The discovery of cancer stem cells over two decades ago underscored the significance of cellular heterogeneity within a given tumor in terms of therapy resistance and disease recurrence (reviewed in [83]). By the mid-2000s, a handful of pioneering cancer biologists who had relied on single-cell studies demonstrated that cancer cells with a highly enlarged nucleus or multiple nuclei (manifestations of mitotic “catastrophe” or “death”) give rise to progeny with stem cell-like properties. In 2001, for example, Erenpreisa and Cragg published a review entitled “Mitotic Catastrophe: A Mechanism of Survival…” in which they concluded that “the features of mitotic death do not simply represent aberrations of dying cells but are indicative of a switch to amitotic modes of cell survival that may provide additional mechanisms of genotoxic resistance” [84].

Cancer cells with extensive nuclear abnormalities (polyploidy, multinucleation, micronucleation) are now referred to as polyploid giant cancer cells (PGCCs) [85,86,87] and have emerged as the root causes of therapy resistance and relapse based on numerous preclinical and clinical studies (reviewed in [69,88]). Like cancer stem cells, PGCCs represent only a small proportion of cells within a solid tumor, thus contributing to intratumor heterogeneity.

In addition to PGCCs, cancer cell dormancy can also represent one or more of the following responses depending on the type of anticancer agent administered and the genetic background of cells (reviewed in [4]): therapy-induced premature senescence (which is often associated with a highly enlarged morphology due to extensive cytoplasmic mass) and the development of drug-tolerant persister cancer cells, radiation-tolerant persister cancer cells, and quiescent cancer cells. Each of these responses is reversible and can lead to the emergence of tenaciously proliferating cancers. It is feasible to assume that more than one these dormancy states can occur in different subsets of cancer cells within a tumor.

For those who are interested in further reading, the aforementioned recent reviews [72,73,74,75,76,77,78,79,80,81,82] have provided a wide range of overviews of the cellular, molecular and clinical heterogeneity in the context of cancer progression, therapy resistance, and recurrence of metastatic disease. It is noteworthy that these reviews do not point out the contribution of therapy-induced responses (anastasis, treacherous apoptosis, PGCCs, cell fusion, etc.) to intratumor heterogeneity discussed herein and in our previous publications (e.g., [2,3,4]). This underscores the tremendous multifactorial nature of tumor heterogeneity (Perhaps the reader might think that this is a rather wishy-washy explanation. I totally agree. How can a therapy-related article by reputable authors disregard the dark sides of apoptosis, PGCCs, senescence, etc.?).

Several questions arise when considering all these therapy-resistance responses, that can underlie tumor diversity, including the following two:

1. Is cancer cell dormancy a greater threat in managing solid tumors or treacherous apoptosis (encompassing anastasis)? Probably the former is a bigger fish to fry based on reasons discussed previously [3,89,90]. For example, judging from tissue culture studies, clinically relevant anticancer exposure (radiation, drugs) triggers cancer cell dormancy but rarely engages regulated cell death [3,89,90,91]. This observation gives credence to the emerging trend of deintensification in cytotoxic cancer therapy [92], which would be expected to minimize the occurrence of side effects as well as regulated cell death and other tumor-repopulating events [4].
2. Given that intratumor heterogeneity was well established over two decades ago [83], why did it take so long for most cancer research community members to appreciate its impact on resistance and relapse? Who knows! Perhaps “in the quest for the next cancer cure, few researchers bother to look back at the graveyard of failed medicines to figure out what went wrong” [93].

## 5. What Are the Reasons for Repeated Failures in Treating Solid Tumor Malignancies?

### 5.1. Most Preclinical Anticancer Studies Generate Clinically Irrelevant Information

The main objective of the various Special Issues of MDPI publications that I have Guest Edited in recent years has been to provide a comprehensive update on the growing complexity of cellular and molecular responses to DNA-damaging anticancer agents in human solid tumors and tumor-derived cell lines (see, e.g., [94]). Most articles published in these collections focused on therapy resistance reflecting genome chaos (e.g., polyploidy), regulated cell death (apoptosis), atavistic reprogramming (unicellular-like stress-resistant traits in cancer), and cell fusion [2,94,95].

**Figure 4:**
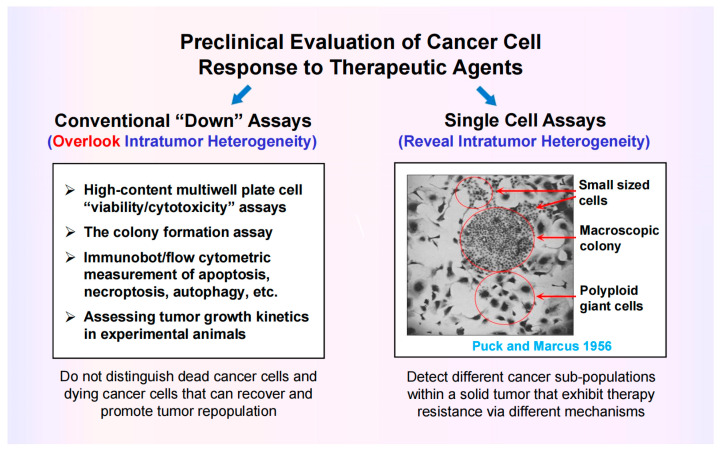
Left: Preclinical assays that are widely used in anticancer drug discovery studies. These so-called “down” assays measure averaged responses of a large number of cells (i.e., overlook intratumor heterogeneity). Old-fashioned microscopy (right), advanced time-lapse microscopy and other single-cell assays are required in order to reveal and study the complexity and heterogeneity that exists within a tumor. The image, showing a remarkable heterogeneity within HeLa cell cultures in response to radiation exposure, was reported 70 years ago (for details, see [69]). Small-sized cells, a macroscopic colony containing small-sized cells, and polyploid giant cells are marked.

The impetus behind leading these Special Issues, as well as writing the current review, has been the following grim reality: these various therapy-resistant and tumor-repopulating responses, as revealed by single-cell analysis, are either overlooked or scored as “death” in ubiquitously used preclinical radiosensitivity and chemosensitivity assays, including those listed in Figure 4 (left).

The danger of relying on these so-called “down” assays (decreased viability, colony forming ability, protein levels, etc.) for measuring cancer cell death has been discussed by us [3,69,95,96,97,98] and others [23,99,100,101]. The conventional in vitro colony formation assay, for example, which is considered the gold standard for measuring cancer cell radiosensitivity and chemosensitivity, determines the ability of a test agent to covert proliferating cancer cells to dormant, tumor-repopulating cells, and not dead cells [95]. In fact, the observation that cancer cells (HeLa) that lose their colony-forming ability in response to stress (exposure to ionizing radiation) remain viable and secrete proliferation-stimulating factors dates back to the 1950s (for details, see [69]) (the image shown in Figure 4 is reproduced from the seminal study reported by Puck and Marcus in 1956 [102]).

With respect to high-content multiwell plate assays, as pointed out by Eastman [99], drug treatment conditions typically used to obtain IC_50_ values (50% inhibitory concentration) “are irrelevant to how drugs are subsequently administered to patients; drugs are selected based on continuous incubation of cells, then frequently administered to the patient as a bolus. Target identification and validation is often performed by gene suppression that inevitably mimics continuous target inhibition” [99]. The same caveats also pertain to assessing cancer cell apoptosis, which is often performed under clinically irrelevant conditions (continuous treatment with high drug concentrations [3,89,99]).

In short, clinically relevant chemotherapy exposure predominantly triggers cancer cell dormancy and not apoptosis. The impact of this statement might not be appreciated without considering values. Some of us have done this math and find the outcome to be shocking. As discussed previously (e.g., [3,69,89]; also see Appendix B), for cisplatin, the maximum tolerated dose (MTD) when administered to live animals is determined to be equivalent to ~10 µM in laboratory studies with cultured cells. In tissue culture studies, drug concentrations required to induce 50% inhibitory effect amount to ~2 µM (way below the MTD) for proliferation arrest (when measured using the colony formation assay and not multiwell plate cell “viability” assays) and >40 µM (>4 times above the MTD) for some indicators of apoptosis.

The basis for this discrepancy was established over 20 years ago. Relatively low concentrations of cisplatin induce sufficient amounts of DNA lesions that inhibit cell proliferation (dormancy via reversible senescence, polyploidy, multinucleation, etc.), whereas very high drug concentrations are needed to damage the cytoplasmic compartments to engage apoptosis.

Such discrepancy for triggering dormancy versus apoptosis has been observed for *all* solid tumor-derived cell lines after exposure to *all* chemotherapeutic drugs that have been tested.

The reader might wonder if thousands of preclinical studies that have relied on apoptosis/cell “viability” assays to measure solid tumor cell fate have generated dangerously misleading and clinically irrelevant information! I think so, and this is why we have been raising red flags for such studies for decades (reviewed in [3,69,89]).

### 5.2. Assessing Cancer Cell Fate Following Exposure to Therapeutic Agents Requires Single-Cell Assays

**Figure 5:**
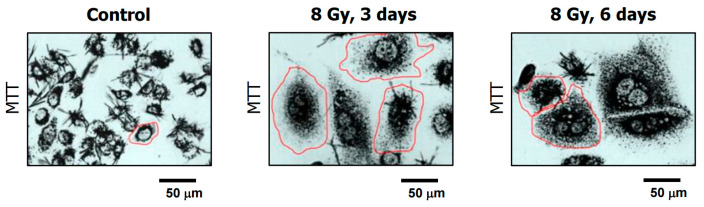
Bright-field microscopy images showing the ability of MCF7 breast cancer cells to convert the MTT reagent to its water-insoluble formazan derivative (dark granules and crystals) before (control) and at indicated times after exposure to ionizing radiation. Images were acquired after cells were incubated with the reagent for ~1 h. The border of some cells is marked for clarity. Reproduced from our open access publication [96].

Details of such assays optimized by us have been published (e.g., [97,103]). These include the single-cell MTT assay which distinguishes dead cancer cells and dying cells that can recover and proliferate. The assay determines the ability of individual cells to metabolize the tetrazolium salt 3-(4,5-dimethylthiazol-2-yl)-2,5-diphenyl-tetrazolium bromide (MTT) to its water-insoluble formazan derivative, which can be visualized as purple intracellular granules and crystals under a light microscope (also see Figure 5). This simple, and yet highly informative, assay circumvents many pitfalls of other viability assays that are based on large dye exclusion (for details, see [97,103]).

### 5.3. The Consequences of Dishonesty in Data Reporting

We have recently discussed the growing complexity of tumor heterogeneity in terms of therapy resistance in various articles, including a review entitled “What are the reasons for continuing failures in cancer therapy? Are misleading/inappropriate preclinical assays to be blamed? Might some modern therapies cause more harm than benefit?” [2]. An important, albeit disturbing topic that we covered in these articles relates to the consequences of dishonesty in data reporting, with numerous publications in major journals containing massaged or falsified results (see, e.g., Section 4 in [2], and Section 4.3 in [69]).

Thanks to the artificial intelligence technology, a significant number of such publications have been (and continue to be) retracted. For example, based on PubMed searches, only in 2025, at least TWENTY p53-related articles and over SIXTY apoptosis-related articles have been retracted (I stopped counting!).

A decade ago, a blog on “Retraction Watch” was published which highlighted five major cancer therapy-related publications from a reputable laboratory that were retracted [104]. In that blog, someone (Todd) raised the following profound question: has anyone, or any organization, “started to audit meta-analyses, systematic reviews, practice guidelines, etc.—to determine the impact of these retractions?”

We have a similar concern regarding thousands of p53/cancer-related articles in which “apoptosis” is used as another word for death. Like the retracted papers, how are these highly biased articles going to impact “meta-analysis, systematic reviews, practice guidelines, etc.”? As stated by Dr. Otis Brawley (previous chief medical officer at the American Cancer Society), the consequences of such sloppiness in biomedical research “are real—and they can be deadly. Patients and their families have bought into treatments that either don’t work, cost a fortune or cause life-threatening side effects” [105].

It is for such reasons that our group has committed to writing articles and leading Special Issues in order to highlight false hypotheses that have derailed cancer research for decades. These include the biological output of p53 signaling discussed below.

## 6. Activation of Wild-Type p53 Signaling Following Clinically Relevant Anticancer Treatment Serves to Suppress (“Treacherous”) Apoptosis

There is a common trend in most reviews on the biological outputs of p53/p21 signaling (e.g., [106,107,108,109,110]). These articles typically provide comprehensive discussion on, e.g., different modes of cell “death” (e.g., apoptosis; cellular senescence; autophagy, ferroptosis), cell metabolism, and the immune system, and the roles played by p53 signaling in regulating these responses. Some reports also discuss the significance of p53 dynamics and cell fate decisions following treatment with ionizing radiation, chemotherapeutic drugs, and small molecule p53 activators such as nutlins. Although the growing complexity of cancer cell response to genotoxic stress has been generally appreciated, these reviews typically focus on the two-armed model of cell fate outcome in response to DNA damage, which was highlighted by Lane in 1992 [111]: namely, p53-dependent cell cycle arrest and apoptosis (e.g., [107]). In this model, p21 is considered to be merely an activator of cell cycle checkpoints.

**Figure 6:**
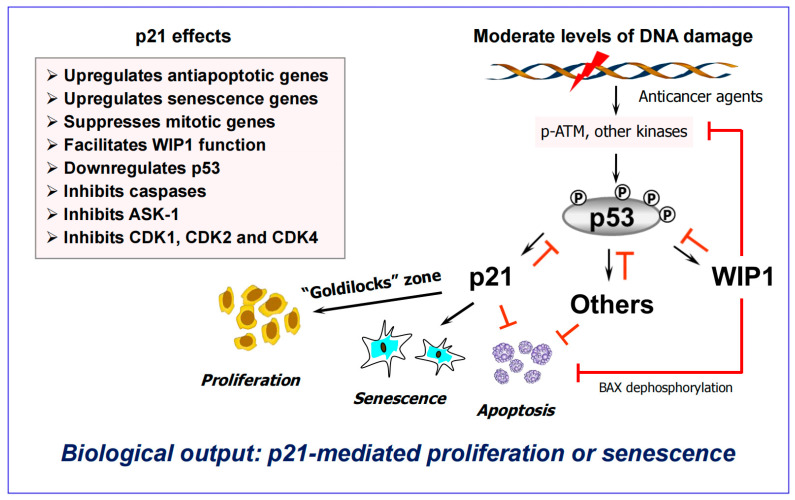
A partial schematic of the DNA damage response network illustrating the importance of negative regulation of p53 by p21, WIP1 (wild-type p53-induced phosphatase 1), and other p53 targets (e.g., MDM2, DNAJB9; not shown) in suppressing regulated cell death. Arrows indicate stimulation and T-shaped lines indicate inhibition. Multiple functions of p21 in the DNA damage response network are indicated.

There are four fundamental issues with this canonical model. First, the biological output of p53-p21 signaling is context-dependent (see below). Second, unlike the conventional wisdom [112,113], apoptosis and senescence are not permanent cell fates. In fact, as recently pointed out by Kandouz [114], it is still uncertain as to what constitutes cancer cell death. Third, clinically relevant anticancer exposure rarely (if at all) engages apoptosis in p53 wild-type solid tumor cells [3,89]. Forth, the landscape of p21 functions has expanded far beyond its classical role as a regulator of cell cycle progression ([115]; also see Figure 6).

It is worth noting that small-molecule activators of p53, such as nutlin-3, have off-target effects. This includes triggering endoreduplication [116,117], which leads to emergence of PGCCs that underlie therapy resistance and tumor repopulation [85,86].

### 6.1. Impact of p53 on Apoptosis Under Non-Physiological Versus Clinically Relevant Conditions

The pro-apoptotic property of wild-type p53, which is often regarded as “indisputable fact” (similar to apoptosis being equal to death), needs to be put into context as follows:

➢ Over two decades ago it was demonstrated that p53 protein levels need to increase above a threshold to induce apoptosis [118,119,120] (we have extensively reviewed these and related discoveries regarding the apoptotic threshold [3,89,121,122]).
➢ Strong p53 activation (above the apoptotic threshold) is observed under non-physiological conditions, such as cell exposure to very high doses of genotoxic agents (cisplatin; UVC) that induce bulky (transcription-blocking) DNA lesions [121].
➢ Under these conditions, bulky lesions prevent transcriptional activation of MDM2 and other p53 negative regulators (e.g., p21, WIP1), resulting in a strong accumulation of p53 protein that triggers apoptosis presumably via its proline-rich region [121].
➢ On the other hand, activation of p53 signaling following exposure to clinically relevant doses of anticancer agents serves to suppress apoptosis and to promote dormancy via premature senescence ([115,121] and Figure 6). Under these conditions, cells rapidly remove bulky lesions from expressed genes through the transcription-coupled subpathway of nucleotide excision repair [121].
➢ By 2008, over forty p53 targets with strong antiapoptotic properties had been identified [123]. These include p21, WIP1 and others (e.g., MDM2, DNAJB9) that form negative regulatory loops with p53 [121,122,123].

In short, activation of p53 signaling under physiological (clinically relevant) conditions appears to function as a strong barrier (“molecular brick wall” [90]) that protects against apoptosis, rather than engaging it. The antiapoptotic property of p53 was originally suggested to reflect its “dark” side [123], but it turns out that preventing treacherous apoptosis (which fuels the oncogenic fire [25]) represents the “bright” side of this important tumor suppressor.

### 6.2. The “Goldilocks Zone” for Cancer Cell Proliferation Following Clinically Relevant Chemotherapy Exposure

**Figure 7:**
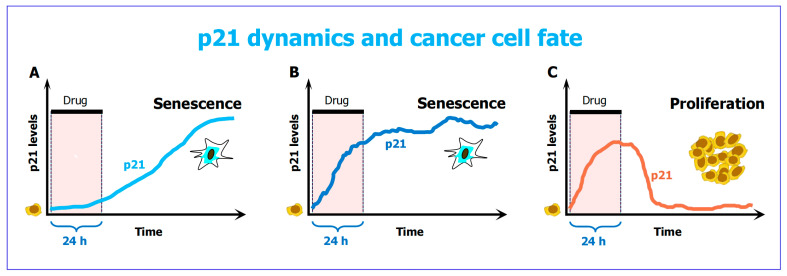
The influence of p21 dynamics on cancer cell fate following a 24-h treatment with the chemotherapeutic drug doxorubicin, reported by Hsu et al. [91]. Cells with either low levels of p21 (**A**) or very high levels of p21 (**B**) following treatment undergo senescence. On the other hand, cells with their p21 at the “just right” level post-treatment (**C**) activate cell cycle checkpoints (to facilitate DNA repair) and resume proliferation. The latter scenario is called the p21 “Goldilocks zone” for proliferation [91].

Time lapse microscopy has revealed that early p21 dynamics predict and shape cellular fate [91]: cancer cells with either low or very high levels of early p21, following chemotherapy exposure, are fated toward premature senescence (Figure 7, panels A and B), whereas cells with intermediate amounts of early p21 exhibit transient cell cycle arrest, decrease their p21 levels and resume proliferation (Figure 7C). The latter scenario has been termed the p21 “Goldilocks zone” for proliferation. Different p21 dynamics and cell fate outcomes in “DNA-repair-proficient cancer cells” (noted in Figure 3) contribute to intratumor heterogeneity.

The study was reported by Hsu et al. [91] in 2019 (also see [68,124]). The experiments were performed with A549 (p53 wild-type) lung carcinoma cells that were treated with 50 nM doxorubicin for 1 day and then incubated with fresh medium (without drug) for 4 days. The authors stressed that this treatment condition, which is known to be clinically relevant, did not engage regulated cell death.

Another pro-survival property of p21 pertains to reversibility of senescence-associated proliferation arrest, giving rise to highly metastatic progeny. This observation was first reported by Igor Roninson’s group over 25 years ago [125,126,127]. The authors concluded that in solid tumor cells, the “re-entry into cell cycle after high-level induction of p21 may serve as a major cause of genetic destabilization that contributes to carcinogenesis and tumor progression” [128]. More recently, other groups have demonstrated that the reversal of senescence can be accelerated following treatment with apoptosis-triggering drugs (e.g., camptothecin; ABT-737) or ectopic expression of caspase 3 [44].

The prosurvival properties of p21 have led to the notion that perhaps selective inhibition of p21 function in cancer cells might result in a favorable patient outcome. This possibility turned out to be untenable. Single-cell studies have demonstrated that loss of p21 (or p53) in cancer cells is permissive for the development of PGCCs [128], which repopulate the tumor (reviewed in [85,86]).

These discoveries need to be taken into consideration when designing therapeutic strategies targeting the p53-p21 pathway.

## 7. Conclusions

Preclinical anticancer studies are designed on the premise that therapy-induced apoptosis and cell proliferation arrest (dormancy) are permanent fates, ultimately leading to cancer cell demise. Accordingly, an enormous effort has been devoted to developing therapeutic strategies centered on apoptosis and durable proliferation arrest (senescence), mediated by wild-type p53 and its downstream effector p21, respectively.

Although apoptosis and senescence are scored as “death” in multiwell plate cell viability and other ubiquitously used preclinical radiosensitivity and chemosensitivity assays, single-cell studies have demonstrated that these responses are reversible, resulting in the emergence of more aggressive cancers (reviewed in, e.g., [4,23,32]). Furthermore, apoptotic cancer cells are known to promote the reversal of proliferation arrest in cancer cells undergoing senescence [44].

### 7.1. Who Would Disregard the Treacherous Side of Apoptosis in Treating Solid Tumors?

The dark side of apoptosis in cancer therapy is highlighted in only a handful of articles (perhaps in no more than a dozen, when excluding publications by our own group). Thus, the majority of publications (articles, reviews, editorials, online blogs) that discuss the challenges and opportunities in implementing precision oncology continue to propose novel apoptosis-stimulating anticancer strategies. The reasons for this serious oversight remain unknown.

Some authors, however, have started to discuss the need for a paradigm shift in the study of cell death in general, and specifically oncology, based on a wealth of preclinical observations (i.e., even after overlooking the aforementioned clinical reports) (e.g., [28,29,30,31]). These preclinical studies argue against the hypothesis proposed over 20 years ago, which is still widely cited (e.g., by the Nomenclature Committee on Cell Death) [35]. Namely, in mammalian cells, “the activation of executioner caspases occurs after the cells are committed to die” [35]. Now we know that there is no point of no return in apoptosis and probably other regulated cell death pathways [29,30]. Thus, “a paradigm shift in the study of cell death is currently occurring” [30].

### 7.2. Call for Contrarian Logic in Cancer Research

I trust that the discoveries highlighted herein and previously [2,3,4] are sufficient to put the following three fundamental questions into perspective:

➢ What is apoptosis? Is it an irreversible mode of cell death based on cell “viability” and other misleading preclinical assays? Or does engaging apoptosis in solid tumors represent a treacherous, tumor-repopulating outcome? (I think it is the latter.)
➢ Is “evading apoptosis” a hallmark of cancer, contributing to tumor progression and therapy resistance, as hypothesized by Hanahan and Weinberg over two decades ago [129]? Or, like normal cells, do cancer cells simply employ the homeostatic process of anastasis to survive after engaging in regulated cell death? (I think it is the latter.)
➢ Is deregulated anastasis a hallmark of cancer? The availability of anastasis markers such as cell surface CD24 expression will hopefully lead to addressing this and other outstanding questions in cancer progression and therapy.

Understanding and counteracting different layers of tumor heterogeneity are paramount for an improved management of cancer in general and metastatic disease in particular [74]. The integration of novel molecular diagnostic technologies aided by machine learning tools offers a promising avenue in this regard [74]. These machine learning tools need to take into consideration the various layers of intratumor heterogeneity discussed herein and previously [2,69,95], which includes non-mutational/non-genetic events such as cell fusion, giving rise to PGCCs that generate tumor-repopulating progeny via amitosis, depolyploidization, and other mechanisms.

The outstanding contributions of pioneering cancer biologists in the complex fields of polyploidy and genome chaos were recently presented in a commentary entitled “Amitotic Cell Division, Malignancy, and Resistance to Anticancer Agents” that we recently published as a tribute to Drs. Kirsten Walen and Rengaswami Rajaraman [130].

## Data Availability

No new data were created or analyzed in this study. Data sharing is not applicable to this article.

## Appendix A

Examples of false hypotheses that have derailed cancer research for decades. For details, see [4].

**Hypothesis**	**Fact**
Wild-type p53 promotes apoptosis.	Activation of the p53-p21 signaling pathway following clinically relevant anticancer exposure serves to suppress (treacherous) apoptosis
In mammalian cells, caspase 3 and other execu-tioner caspases are activated after the cells are committed to die.	Caspase 3 fuels the oncogenic process and there are no points of no return in various cell death pathways
The terms “apoptosis” and “death” can be used interchangeably.	Cancer cells can recover from late stages of apoptosis via anastasis, giving rise to aggressive variants
In vitro “live/dead” assays generate clinically relevant information.	Loss of cell membrane integrity, detected by vital dye assays, is often transient. Cells have repair mechanisms that can rapidly reseal breaches.
Cancer cells evade apoptosis to survive anti-cancer treatment.	High-grade cancers with poor prognosis contain relatively high levels of apoptotic cells.
Apoptosis is a tumor suppression mechanism.	Apoptosis promotes tumorigenesis via phoenix rising, nu-clear expulsion, and other mechanisms.
Identifying drugs that reinstate or promote apoptosis could improve patient outcomes.	In addition to triggering treacherous apoptosis, this out-dated, and yet widely popular, strategy will not address the impact of amitosis and various other non-genetic tu-mor-repopulating mechanisms.

## Appendix B

Discrepancies in chemotherapeutic drug concentration requirements to induce proliferation arrest (dormancy) and apoptosis in solid tumor-derived cell lines. For details, see [3].

➢ Cisplatin concentrations between 5 and 10 µM used in cell-based studies are determined to be comparable to concentrations that may be achieved in tumor/tissues of treated patients and tumor-bearing laboratory animals. Higher concentrations result in severe side effects in animal studies.
➢ In HCT116 colon carcinoma cells, the IC_50_ (half-maximum inhibitory concentration) values for triggering cancer cell dormancy (durable, but reversible proliferation arrest) is ~2 µM, which is below the maximum tolerated dose (MTD) of ~10 µM.
➢ The average IC_50_ value for engaging apoptosis is ~40 µM (and can be as high 100 µM), which is way above the MTD.
➢ The basis for this discrepancy is known. Relatively low concentrations of cisplatin induce sufficient amounts of DNA lesions that inhibit cell proliferation, whereas very high drug concentrations are needed to damage the cytoplasmic compartments to engage apoptosis.
➢ Such discrepancy in IC50 values for triggering inhibition of cell proliferation (dormancy) and engaging apoptosis has been observed for various solid tumor-derived cell lines after exposure to all other chemotherapeutic drugs that have been tested, including oxaliplatin, paclitaxel, and doxorubicin.
